# A Systematic Review on Subjective Cognitive Complaints: Main Neurocognitive Domains, Myriad Assessment Tools, and New Approaches for Early Detection

**DOI:** 10.3390/geriatrics10030065

**Published:** 2025-05-09

**Authors:** Felipe Webster-Cordero, Lydia Giménez-Llort

**Affiliations:** 1 Department of Psychiatry and Forensic Medicine, School of Medicine, Universitat Autònoma de Barcelona, E-08193 Barcelona, Spain; 2 Hospital Santa Inés, Neuropsychology Service, Av. Daniel Córdova T, 2-67 y Agustín Cueva, Cuenca 010107, Ecuador; 3 Institut de Neurociències, Universitat Autònoma de Barcelona, E-08193 Barcelona, Spain

**Keywords:** subjective cognitive complaints, dementia, neuropsychological assessment, neuropsychological test

## Abstract

**Background/Objectives:** Neuropsychological testing is key in defining cognitive profiles at early stages of dementia. More importantly, the detection of subtle cognitive changes, such as subjective cognitive complaints (SCCs), an understudied phenomenon, is critical for early detection and preventive interventions. **Methods:** This systematic review analyzes the empirical data on the cognitive domains and neuropsychological tests used in studies addressing SCC in the last 15 years (2009–2024). **Results:** A selection of 15 papers with exploratory, cross-sectional, and prospective scope in this field was obtained from PubMed and Embase databases. They used screening tests (17%) and a broad spectrum of neurocognitive domains. Yet, we identified three main targeted cognitive domains: executive functions (28%), language (17%), and memory (17%). Myriad assessment tools were also applied, but the most commonly used was a set of eight tests: Mini-mental Scale Examination (MMSE), Trail Making Test (A-B), Stroop test, Digit span test (DST), Semantic and Phonological fluency test, Rey Auditory Verbal Learning Test (RAVLT), Weschler Memory Scale (WMS), and Boston Naming Test (BNT). New approaches involved including the Geriatric Depression Scale (GDS) and self/informant reports. **Conclusions:** Despite scarce agreement in the assessment protocols, the identification of early neurocognitive symptoms to objectivate the SCC phenomenon envisions a broad field of research.

## 1. Introduction

To date, there has been an increase in the number of people attending neurological and/or neuropsychological consultations due to the presence of subjective cognitive complaints (SCCs). Although this or similar terms, such as subjective cognitive decline (SCD), are not included in international diagnostic manuals ICD-11 or DSM-5, SCC has gained significant relevance over the past decade to describe a condition characterized by a persistent self-perception of reduced cognitive performance beyond what is typical, without objective evidence, following standardized clinical assessment [1]. Its importance in the clinical field lies in its potential relationship to subsequent progression to disorders such as mild cognitive impairment (MCI) and dementia [1,2,3]. Thus, it is estimated that within one year, SCC may progress to MCI in 6.6% of cases and to dementia in 2.3%; over four years, this progression increases to 24.4% for MCI and 10.9% for dementia [4]. Consequently, research on this phenomenon and its potential relationship with the preclinical onset of certain neurodegenerative diseases has grown in the last decade.

Clinical neuropsychology has provided a broad perspective on cognitive changes associated with various neurological conditions to facilitate their diagnosis, serving as a screening before biological markers or other tests can be applied. In the dementia continuum, neurocognitive evaluation has been incorporated into the protocols of multidisciplinary teams worldwide, from preclinical and prodromal to advanced stages [5,6]. Based on clinical judgment and the analysis by complementary laboratory and neuroimaging tests, the professional team determines a final diagnosis to implement specific treatments that can be also monitored [7]. Consequently, neuropsychological assessment has become indispensable in clinical evaluations because of its contribution to identifying existing cognitive deficits to support differential diagnoses, clinical management, and forensic utility [5,6]. In this context, specific neuropsychological assessment for patients with cognitive complaints and suspected neurodegenerative diseases should also complement routine medical studies [8]. However, as shown by clinical studies addressing early detection of subtle cognitive changes, many neuropsychological tests are used, ranging from screening tests to domain-specific tests. Most importantly, there is no consensus on the types of tests to be applied in clinical evaluations, with the choice of tests varying depending on the study [9]. Since SCC remains a poorly explored phenomenon, yet is critical for early detection and preventive interventions, this review aims to systematically analyze the empirical data on the neuropsychological tests used in the different studies addressing subjective cognitive impairments.

## 2. Methodology

**Figure 1:**
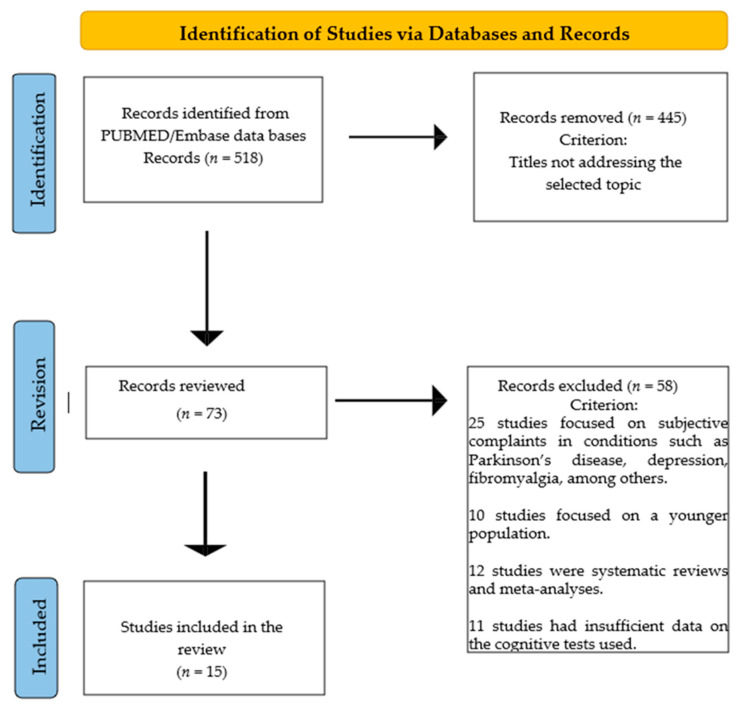
Flow chart of the systematic review neuropsychological evaluation of SCC (2009–2024).

The objective of this review is to systematically analyze empirical data on neuropsychological tests used in various studies addressing the topic of SCC. This systematic review was registered at the PROSPERO 2025 CRD420251029323. PRISMA method guidelines [10] were used to ensure its proper execution (Figure 1). 

### 2.1. Systematic Search (Databases, Descriptors, Search Formulas)

Since the number of scientific publications interested in SCC increased at the end of the first decade of this millennium, the studies of the last 15 years (2009–2024) on the selected topic were analyzed using the PubMed and Embase databases as the primary tools. The terms “neuropsychological evaluation”, “cognitive assessment”, “neuropsychological assessment”, “subjective cognitive decline”, and “subjective cognitive complaints” used in the search with the Booleans “AND” and “OR” yielded 518 results as potential sources for analysis. Inclusion and exclusion criteria were then established. 

### 2.2. Inclusion Criteria

- Empirical research on neuropsychological tools used in the study of subjective cognitive complaints.
- Studies published in the last 15 years with a population aged 60 or older with subjective cognitive complaints.

### 2.3. Exclusion Criteria

- Studies addressing neuropsychological tests for subjective cognitive complaints in other clinical contexts (pathologies or neurologic/psychiatric diseases that may also involve SCC).
- Research exploring neuropsychological assessments in mild cognitive impairment and advanced stages of dementia.

### 2.4. Flow Chart

Following these criteria, 73 studies were initially selected based on their titles. After reviewing their abstracts, 58 studies were excluded because they did not meet the specific objective of this review. Finally, 15 articles were selected for review because they focused on the study of cognitive performance in individuals with subjective cognitive complaints. 

## 3. Results

### 3.1. Most Relevant Data from the Studies Included in This Review

Table 1 highlights a summary of the most relevant data from the 15 selected clinical studies. The studies used longitudinal, follow-up experimental, and cross-sectional descriptive designs. All studies included neuropsychological assessments as a critical component in analyzing the clinical profiles of their participants.

### 3.2. Participants and Sociodemographic Variables

Analyzing the overall data, a total sample of 589,008 participants is included from Western countries (Australia, Central and Southern Europe, North and South America) and Eastern countries (Korea and China). Sociodemographic variables include gender (with a women: men ratio of 3:1), age (average 65.4 years), and years of education (average 9.9 years).

### 3.3. Neuropsychological Domains

Despite the significant heterogeneity in the methodology used in each study and the complexity of clinical assessment in these preclinical phases, current studies on SCC are aligned to consider it as a clinical entity with its characteristics that can be objectified through formal assessment protocols and be a stage prior to MCI and dementia [4,26].

**Figure 2:**
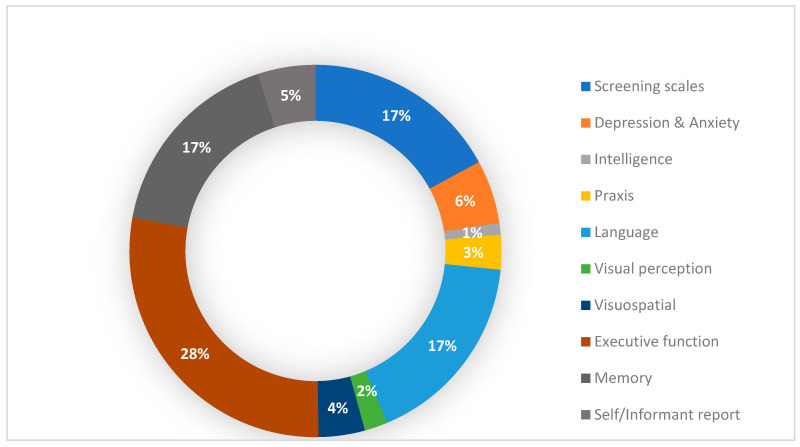
Neuropsychological Domains Assessed in Subjective Cognitive Complaints. Myriad neuropsychological domains assessed in people with subjective cognitive complaints. The number of works addressing the domains is shown in percentages. Domains assessed according to the respective authors: Screening scales. Tests for depression and anxiety, intelligence, praxis, language, visual perception, visuospatial perception, executive function, and memory. Self and informant report.

Different cognitive and behavioral domains were assessed using various neuropsychological tools. As illustrated in Figure 2, a significant number of studies applied tests for executive functions (28%), screening tests (17%), and tests to assess language and memory processes (17%). Other cognitive domains were assessed to a lesser extent (depression/anxiety—6%, self-reports/report informants—5%, visuospatial processes—4%, praxis—3%, visual perceptual processes—2%, intelligence—1%).

### 3.4. Neuropsychological Tests

On the other hand, the most commonly used neuropsychological tests in the different studies were the MMSE as the main screening test (77%), the TMT A-B, Stroop test and the DST for executive performance (62%, 38% and 38% respectively), Semantic and Phonological fluency tests and the BNT for language (38% and 31% respectively), the RAVLT (31%) and the WMS (23%) as memory tests, and the GDS (22%) for the assessment of anxiety and depression symptoms (Table 2).

## 4. Discussion

The preclinical and prodromal stages of dementia are associated with subtle changes in various cognitive processes beyond memory, which were initially linked to neurodegenerative diseases [5]. Employing neuropsychological tests as part of care protocols for patients with early cognitive difficulties has become an essential tool for the early diagnosis of potential neurodegenerative conditions, such as dementia. However, identifying tools sensitive enough to assess neurocognitive processes—particularly in a subjective phenomenon like SCC—is complex and demands extensive analysis [1,6,27,28].

In this review, it is found that various authors employed neuropsychological screening tests to assess people with SCC. In some instances, screening was followed by specific tests or scales targeting different cognitive domains, including memory, language, perception, visuospatial function, praxis, and executive function [11,12,13,14,15,16,17,18,19,20,21,22,23,24,25]. Among the limitations observed are the methodological heterogeneity among studies, the fact that some tools can be used to assess more than one domain, the complexity of clinical assessment during these preclinical phases, along with the lack of agreement on selection criteria. Despite these challenges, current studies on SCC aligned to consider its characteristics can be objectively evaluated through formal assessment protocols, positioning SCC as a potential stage prior to MCI and dementia [4,26].

The validity of screening tests in identifying preclinical and prodromal signs of dementia is often insufficient. While there are screening tools that may indicate potential issues, they are not definitive. Nonetheless, some studies in this review suggested [11,12,13,14,15,16,17,19,20,23,25] that the Mini-Mental State Examination (MMSE), the Montreal Cognitive Assessment (MoCA), and the Alzheimer’s Disease Assessment Scale–Cognitive (ADAS-cog) were primarily used. Some authors believe that the MMSE may be accurate for detecting Alzheimer’s disease; however, they recommend using the Montreal Cognitive Assessment (MoCA) for detecting MCI, as it is a more sensitive test for measuring early cognitive changes [29,30].

In contrast, it has always been argued that tests such as the Mini-Mental State Examination (MMSE), among other screening tools, have not proven to be sufficiently sensitive for assessment in primary care. The authors further discuss that other factors, such as administration time or individual circumstances (educational level, sensory problems, and others), may interfere with applying basic tests, failing to identify specific cognitive issues. Kueper et al. [31], on the other hand, suggest that the ADAS-cog is not an optimal tool for detecting subtle pre-dementia changes and that it should be used in parallel with other tests that more accurately assess memory processes, executive functions, and daily living activities. In fact, in our recent clinical study in an at-risk Sub-Saharan diabetic population, the use of a 6-CTI scale in parallel to MMSE was shown to be more sensitive to unveiling the existence of cognitive deficiencies in the subgroup of the low-educated diabetic female population [32].

The use of specific neurocognitive tests is essential in clinical assessment. Most studies (11 out of 15) used screening tests, and also most of them (10 out of 15) applied other complementary tests targeting different cognitive domains [11,13,14,16,17,18,20,22,23,24]. The studies also pointed out the relevance of using specific neurocognitive tests and considering formal informant reports that can provide sufficient information to the clinician to make more accurate diagnosis [33]. However, the assessment of different cognitive domains poses a challenge in determining early cognitive impairment in neurodegenerative diseases. Although the studies included in this review used several specific cognitive tests, there was no consensus on which tests are crucial for implementation and which key cognitive process should be evaluated. Despite this fact, as can be seen, several of the studies on SCC performed an in-depth assessment of mnestic, linguistic, and executive processes. This will be in agreement with measures of delayed recall being considered the best neuropsychological predictors of conversion from mild cognitive impairment to Alzheimer’s disease [34,35], as well as certain linguistic components [36,37]. On the other hand, the literature also suggests that the assessment of executive functions such as working memory, attentional control, planning, and others could be a critical factor in the identification of possible pathological signs in the preclinical stages of dementia [38,39].

The terminology used to refer to subjective complaints of patients in studies determining their possible relationship with MCI and dementia is heterogeneous. This can be probably considered the main limitation in this field. Subjective cognitive complaints are still a phenomenon, a “non-clinical” entity for which different terms such as “subjective cognitive complaints”, “subjective cognitive decline”, “subtle cognitive impairment”, “pre-MCI”, or “pre-AD” are used. Their equivalence corresponds to the fact that all studies define this phenomenon as the preclinical stage of various conditions that can transform into MCI or dementia. To solve this issue, in the present review, the verbatim from each study has been taken. For instance, in Valech’s study [17], the term “pre-AD” refers to a preclinical stage of Alzheimer’s disease, where there is no objective cognitive impairment. In this particular study, in addition to identifying amyloid β (Aβ) in these stages, the authors determined that subjects may already show subjective decline. They administered a cognitive complaints questionnaire to patients and their families so they could determine the importance of studying subjective complaints at the executive and linguistic levels in the preclinical stages of Alzheimer’s disease.

When referring to the new approaches, it is interesting to note that a small number of studies (4 out of 15) included scales to assess emotional symptoms (depression or anxiety) [13,14,16,17]. Assessing the emotional domain in older adults with cognitive complaints is crucial; the presence of depressive and anxious symptoms in patients with subjective cognitive complaints is of clinical interest because of their potential association with cognitive impairment and progression to neurodegenerative diseases. The coexistence of depression and/or anxiety with subjective cognitive decline is associated with an increased risk of developing dementia [2,39,40,41]. Therefore, early identification of these symptoms is a crucial component in understanding the subtle emotional changes that may also precede this disease.

In addition, the use of self-report scales and informant reports seemed to complement the clinical assessment significantly. Some of the reviewed studies used this tool in their studies [13,17,20,21,23]. The literature indicates that incorporating these tools into cognitive impairment evaluations is essential, as they provide valuable insights into how daily activities may be perceived by individuals and/or their informants [42,43,44].

Interestingly, behavioral and personality changes do not seem to be of interest in the reviewed studies on SCC. This idea differs from other authors who argue that behavioral changes may be an early signal during the transitional phases between mild cognitive impairment and certain forms of dementia, such as Alzheimer’s disease [45,46].

At last, the limited number of studies and the lack of detailed information about the cultural and educational backgrounds of the participants contribute to heterogeneity and pose significant methodological limitations. Most tests have been designed for Western populations, making the linguistic, educational, and literacy levels of participants crucial for their applicability. Currently, in addition to the existing population, the number of individuals with cognitive impairments from non-Western backgrounds, particularly in lower-middle-income economies, has increased. This trend underscores the need for a methodology that is more valid for individuals with lower educational levels and that allows for the cross-cultural applicability of the instruments used [32,47,48].

Overall, the various studies reviewed here exhibit significant complexity and heterogeneity when analyzing cognitive performance in patients with SCC. This highlights the urgent need for more evidence regarding the tools to be used in such early stages of cognitive impairment, understanding their reliability and validity, and the multiple variables that may interfere with the results of a standardized and formal evaluation [49]. In addition, studies should accurately determine the characteristics of the SCC phenomenon and emphasize qualitative rather than quantitative analysis of the neuropsychological tests applied, so that preventive/therapeutic intervention protocols may be useful for early care of these conditions [50].

## 5. Conclusions and Future Directions

Clinical attention to subjective cognitive complaints and the tools used to objectify them through the application of a precise neuropsychological evaluation remains challenging for mental health professionals. The heterogeneity of individual characteristics has hindered the availability of tools sensitive enough to detect the initial, often subtle, changes in cognitive performance during the preclinical stages of cognitive decline in older adults. On the other hand, the lack of homogeneity in the protocols used highlights the still uncertain path of clinical tools to unveil the possible subtle changes that occur in preclinical stages of neurodegenerative conditions and to which patients’ complaints refer.

The present review identifies that the different studies on SCC agree on the need to perform a screening and specific assessment of cognitive domains, targeting the executive, linguistic, and mnemic components. When analyzing the methodology, there was no agreement on the neurocognitive tests that should be applied to this clinical population with subjective complaints. Yet, the quantitative analysis of their representation in the different studies can give an estimation of clinical common criteria (cognitive domains to be addressed) and the most used tests to do so. Thus, the summary table identifies a set of “most used” tests for these three domains. According to the most recent works, any evaluation protocol should also be complemented with scales or questionnaires that assess emotional aspects and personality. There is sufficient evidence showing that some neurodegenerative conditions can be characterized by behavioral and mood changes, sometimes isolated from cognitive issues, and these may be part of the evolution of the incipient clinical condition. Similarly, formal self-report questionnaires, supplemented by those of informants, can provide valuable information to determine the impact of cognitive complaints on daily activities and provide greater precision when assessing individuals’ functionality.

Finally, when addressing the complexity and limitations of neuropsychological assessment in the preclinical stages of dementia, it is essential to conduct studies that accurately unify the criteria for clinical characteristics and possible protocols to be used for the management of subjective cognitive impairment, differentiating them from those applied to mild cognitive impairment. Furthermore, it is crucial not to overlook the complex task of qualitative clinical assessment in any neuropsychological performance study, which should be the subject of future research.

## Figures and Tables

**Table 1:** Studies about neuropsychological domains and assessment tools in subjective cognitive complaints.

Authors [Reference] Country	Sample Subjects’ Diagnosis (Gender Ratio) [Mean Age] {Mean Years of Education}	Domains Assessed	Tests Used
van Harten et al. [11] The Netherlands	132 participants with SCC (56 W:76 M) [61.4] {6}	Screening scales Depression/anxiety scales Intelligence Praxis Language Visual perception Visuospatial Executive function Memory Self/Informant report	MMSE - - - Semantic fluency - - TMT A-B, DST VAT, RAVLT -
Toledo et al. [12] USA	522 participants (253 W:269 M): 307 CN subjects (138 W:169 M) [73.9] {-} 71 subjects SCC (25 W:46 M) [71.6] {-} 51 subjects executive SCI (21 W:30 M) [77.3] {-} 66 subjects memory SCI (49 W:17 M) [75] {-} 27 subjects multi-domain SCI (20 W:7 M) [78] {-}	Screening scales Depression/anxiety scales Intelligence Praxis Language Visual perception Visuospatial Executive function Memory Self/Informant report	MMSE, ADAS - - - - - - - - -
Seo et al. [13] Korea	265 participants (178 W:79 M): 188 CN subjects (120 W:60 M) [71.94] {9.69} 77 subjects pre-DCL (58 W:19 M) [72.64] {9.64}	Screening scales Depression/anxiety scales Intelligence Praxis Language Visual perception Visuospatial Executive function Memory Self/Informant report	MMSE, SNSB GDS - RCFT CPFT, K-BNT - RCFT TMT A-B, DST, Stroop test, SVLT SMCQ (informant report)
Verfaillie et al. [14] The Netherlands	233 participants SCD (107 W: 125 M) [62.82] {5.32}	Screening scales Depression/anxiety scales Intelligence Praxis Language Visual perception Visuospatial Executive function Memory Self/Informant report	MMSE GDS - - Semantic fluency - - TMT A-B, DST, Stroop test RAVLT, VAT -
Bae et al. [15] South Korea	1442 participants (-) [-] {-}: 1088 HC subjects (-) [-] {-} 354 SCC (-) [-] {-}	Screening scales Depression/anxiety scales Intelligence Praxis Language Visual perception Visuospatial Executive function Memory Self/Informant report	MMSE - - - - - - K-DRS (Inhibition/Perseveration) - -
Viviano et al. [16] USA, The Netherlands	83 participants (51 W:32 M): 35 adults with SCI (22 W:13 M) [68.5] {-} 48 adults without SCI (29 W:19 M) [67.08] {-}	Screening scales Depression/anxiety scales Intelligence Praxis Language Visual perception Visuospatial Executive function Memory Self/Informant report	MMSE, BFI GDS, BDI - - - - - - WMS -
Valech et al. [17] Spain	68 normal subjects (46 W:22 M): 52 HC (33 W:19 M) [63.87] {11.96} 16 pre-AD (13 W and 3 M) [66.5] {9.56}	Screening scales Depression/anxiety scales Intelligence Praxis Language Visual perception Visuospatial Executive function Memory Self/Informant report	MMSE HADS - - BNT, BDAE, Semantic fluency VOSP - TMT A, Stroop test MAT, FCSRT-IR SCD-Q
Pérez et al. [18] Spain	195 participants SCD (121 W:74 M) [65.71] {14.94}	Screening scales Depression/anxiety scales Intelligence Praxis Language Visual perception Visuospatial Executive function Memory Self/Informant report	- - WAIS - Semantic fluency, Phonological fluency - - TMT A-B, RSCS-BADS, AI-SKT - -
Kim et al. [19] Korea	1442 participants (886 W:556 M) [≥65 years]: 1088 HC subjects (642 W:446 M) {5.66} 354 SCC subjects (244 W:110 M) {3.33}	Screening scales Depression/anxiety scales Intelligence Praxis Language Visual perception Visuospatial Executive function Memory Self/Informant report	MMSE-KC - - - - - - K-DRS (Inhibition/Perseveration) - -
Hao et al. [20] China	615 subjects SCD *plus* (378 W:228 M) [-] {-}	Screening scales Depression/anxiety scales Intelligence Praxis Language Visual perception Visuospatial Executive function Memory Self/Informant report	MoCA - - CDT Semantic fluency - - TMT B AVLT-H SCD-Q
Lee et al. [21] Korea	579.710 subjects (313.399 W:266.311 M) [66] {11.6}: 357.654 subjects Non-SCD 222.056 subjects SCD	Screening scales Depression/anxiety scales Intelligence Praxis Language Visual perception Visuospatial Executive function Memory Self/Informant report	- - - - - - - - - (KDSQ-P)
Numbers et al. [22] Australia	873 subjects SCD (480 W:392 M) [78.65] {-}	Screening scales Depression/anxiety scales Intelligence Praxis Language Visual perception Visuospatial Executive function Memory Self/Informant report	- - - - BNT, Semantic fluency, Phonological fluency - Block Design Digit Symbol Coding, TMT A-B, WMS, RAVLT, Benton Visual Test -
Garrido et al. [23] Spain	136 participants (67 W:59 M): 28 young adults with SCC (17 W:11 M) [21] {-} 37 young adults without SCC (16 W:11 M) [23] {-} 32 older adults with SCC (18 W:14 M) [63] {-} 39 older adults without SCC (16 W:23 M) [65] {-}	Screening scales Depression/anxiety scales Intelligence Praxis Language Visual perception Visuospatial Executive function Memory Self/Informant report	MMSE - - RCFT Semantic fluency, Phonological fluency - - TMT A-B, Stroop test, DST, IGT FCSRT MFE-30
Oliver et al. [24] USA	3019 healthy older adults 831 with SCD (635 W:196 M) 2188 without SCD (1660 W:528 M) [73.6] {14.74}	Screening scales Depression/anxiety scales Intelligence Praxis Language Visual perception Visuospatial Executive function Memory Self/Informant report	- - - - BNT NC PM, Line Orientation DST, SDMT, Stroop test WMSR, EBS, WLMR -
Morrison et al. [25] Canada	273 participants: 97 with SCD (36 W:61 M) 176 without SCD (90 W:86 M) [72.97] {16.66}	Screening scales Depression/anxiety scales Intelligence Praxis Language Visual perception Visuospatial Executive function Memory Self/Informant report	ADAS-13, MMSE, MoCA - - - - - - - - -

Abbreviations: W: women. M: men. SCC: subjective cognitive complaints. SCD: subjective cognitive decline. SCI: subtle cognitive impairment. HC: Healthy control. MMSE: Mini-mental Scale Examination. ADAS: Alzheimer’s Disease Assessment Scale. MoCA: Montreal cognitive assessment. WAIS: Wechsler Adult Intelligence Scale. RCFT: Rey–Osterrieth complex figure test. BNT: Boston Naming Test. K-BNT: Korean version of the Boston Naming Test. CDT: Clock Drawing Test. TMT (A-B): Trail Making Test. RAVLT: Rey Auditory Verbal Learning Test. DST: Digit span test. VAT: Visual Association Test. SNSB: Seoul Neuropsychological Screening Battery. GDS: Geriatric Depression Scale. CPFT: Categorical and phonemic fluency tests. SVLT: Seoul Verbal Learning Test. SMCQ: Subjective Memory Complaints Questionnaire. K-DRS: Subscale of the Korean version of Matts Dementia Rating Scale. BFI: Big Five Inventory. BDI: Beck Depression Inventory. WMS: Weschler Memory Scale. HADS: Hospital Anxiety and Depression Scale. BDAE: Boston Diagnostic Aphasia Examination-III Edition. VOSP: Visual Object and Space Perception Battery. MAT: Memory Alteration Test. FCSRT-IR: Free and Cued Selective Reminding Test. SCD-Q: Subjective Cognitive Decline Questionnaire. RSCS-BADS: Rule Shift Cards subtest. AI-SKT: Automatic inhibition subtest. MMSE-KC: Mini-mental Mental State Examination Korean version. K-DRS: Korean rating scale. AVLT-H: Auditory Verbal Learning Test Hua Shan. IGT: Iowa Gambling Task. FCSRT: Free and Cued Selective Reminding Test. MFE-30: Memory Failures of Everyday Questionnaire. NC: Number Comparison. PM: Progressive Matrices. SDMT: Symbol Digital Modalities Test. WMSR: Wechsler Memory Scale-Revised. EBS: East Boston Story, WLMR: Word List Memory Recall and Recognition. KDSQ-P: Pre-screening Korean Dementia Screening Questionnaire.

**Table 2:** Main tools used to assess cognitive domains in people with subjective cognitive complaints.

Domains	Tool	*n*	%
Screening	MMSE	10	77
Executive function	TMT A-B	8	62
	Stroop test	5	38
	DST	5	38
Language	Semantic and Phonological fluency test	5	38
	BNT	4	31
Memory	RAVLT	4	31
	WMS	3	23
Depression and Anxiety	GDS	3	23

Abbreviations: MMSE: Mini-mental Scale Examination. TMT (A-B): Trail Making Test. DST: Digit span test. BNT: Boston Naming Test. RAVLT: Rey Auditory Verbal Learning Test. WMS: Weschler Memory Scale. GDS: Geriatric Depression Scale.

## Data Availability

Not applicable.
